# Patterns of collaboration in complex networks: the example of a translational research network

**DOI:** 10.1186/1472-6963-14-225

**Published:** 2014-05-20

**Authors:** Janet C Long, Frances C Cunningham, Peter Carswell, Jeffrey Braithwaite

**Affiliations:** 1 Centre for Clinical Governance Research, Australian Institute of Health Innovation, Faculty of Medicine, University of New South Wales, Kensington 2052, Australia; 2 Centre for Primary Health Care Systems Research, Menzies School of Health Research, Level 1, 147 Wharf Street, Spring Hill, QLD 4000, Australia; 3 School of Population Health, University of Auckland, Auckland, New Zealand

**Keywords:** Network theory, Collaboration, Translational research, Proximity, Brokerage, Health, Silos, Interorganisational alliances, Collaboratives for leadership in applied health research and care (CLAHRCs)

## Abstract

**Background:**

This paper examines collaboration in a complex translational cancer research network (TRN) made up of a range of hospital-based clinicians and university-based researchers. We examine the phenomenon of close-knit and often introspective clusters of people (silos) and test the extent that factors associated with this clustering (geography, profession and past experience) influence patterns of current and future collaboration on TRN projects. Understanding more of these patterns, especially the gaps or barriers between members, will help network leaders to manage subgroups and promote connectivity crucial to efficient network function.

**Methods:**

An on-line, whole network survey was used to collect attribute and relationship data from all members of the new TRN based in New South Wales, Australia in early 2012. The 68 members were drawn from six separate hospital and university campuses. Social network analysis with UCInet tested the effects of geographic proximity, profession, past research experience, strength of ties and previous collaborations on past, present and future intended partnering.

**Results:**

Geographic proximity and past working relationships both had significant effects on the choice of current collaboration partners. Future intended collaborations included a significant number of weak ties and ties based on other members’ reputations implying that the TRN has provided new opportunities for partnership. Professional grouping, a significant barrier discussed in the translational research literature, influenced past collaborations but not current or future collaborations, possibly through the mediation of network brokers.

**Conclusions:**

Since geographic proximity is important in the choice of collaborators a dispersed network such as this could consider enhancing cross site interactions by improving virtual communication technology and use, increasing social interactions apart from project related work, and maximising opportunities to meet members from other sites. Key network players have an important brokerage role facilitating linkages between groups.

## Background

Networks in healthcare are promoted as a way to foster collaboration through the organisation of people across facilities, sites and disciplines [1]. Yet our understanding of the structural features and processes within such networks and how they contribute to network outcomes is limited [2,3]. Close-knit groups of people (clusters) within networks can be strong and positive, or negative and counterproductive structures. “Silo” is a generally negative term for a cluster implying insularity, introspection and resistance to change; silos have been shown to hinder collaboration [4-9].

Clusters can also be strong, resilient and highly productive groupings and may hold highly specialised knowledge and expertise [10,11]. Linking clusters together helps communication and information to permeate throughout the whole network, and can also produce innovative ideas through cross-fertilization of the expertise and knowledge held in each [12]. Quantitative examination of these groups and gaps is rare, especially in healthcare settings [5].

The purpose of this paper is to examine factors that influence collaboration among the members of a new translational research network (TRN), affecting past collaborative ties, as well as current and future collaborations. Understanding more about these patterns, especially the gaps, clusters or barriers between members, responds to the identified gap in the literature around the paucity of quantitative studies examining structural features of health networks. It also offers a practical contribution by identifying how network leaders can help leaders of networks to manage subgroups, promote strategic partnering and overall connectivity. This study forms part of a more extensive study looking at network structure and key players and their effect on network outcomes [13-15].

Collaboration is theoretically and conceptually a complex organisational characteristic [16-21]. It has been defined by Wood and Gray [20] as an interactive process between autonomous stakeholders who can see different aspects of a common problem. Collaboration between researchers and clinicians is considered essential in translational research. Translational research undertakes the crucial role of moving biomedical discoveries out of the highly controlled laboratory environment and applying it in the messiness and complexity of actual patient and clinical service realities [22-24]. Expertise and understanding from both arenas through collaboration are necessary to work out ways to make this happen. Yet, the research and clinical domains seem divided by ever-widening gaps of increased specialisation, complexity and sophistication on either side [25,26].

TRNs are a strategy to facilitate translation: setting up an administrative structure to provide funding, project officers and shared resources, and a social-professional structure that can maximize opportunities for collaboration, innovation and knowledge transfer across different disciplines, organisations, sites and specialties [25,27,28]. They manifest in similar ways with different titles: as Collaboratives for Leadership in Applied Health Research and Care (CLAHRCs) in the UK and as interorganisational alliances awarded funded by the Clinical and Translational Science Awards in the USA. Potential partners abound in such networks but clusters and gaps in the organisation of the network can affect the likelihood of collaboration [29,30]. We use a social network approach to examine the collaborative ties among TRN members, forming graphs of linkages (sociograms) that can be analysed for patterns [13].

### Setting

The translational research network (TRN) that is the subject of this project was established in late 2011 as a result of a competitive process for external funding. The membership of 68 cancer clinicians and researchers was drawn from six hospital and university campuses in New South Wales, Australia and a network director, manager and project officers were appointed.

The overarching goal of the TRN is “taking science to practice” and is focused on putting clinically proven knowledge of disease processes, diagnostic or treatment techniques into routine clinical practice and health decision-making [31]. The network provided access to funding for short-term projects, shared databases and facilities, and support from project officers and translational research fellows.

The TRN is embedded in a complex interorganisational network of long-standing research and teaching arrangements. TRN activities and funded projects do not displace existing and ongoing research such as National Health and Medical Research Council (NHMRC) funded projects. At its inception, the TRN was already a collaborative effort with core group members who prepared and submitted the proposal to the funding body.

### Patterns of collaboration

Clusters have been found within networks based on homophily. Homophily is defined by Rogers [32] as “the extent to which two or more individuals who interact are similar in certain attributes”. This similarity means they communicate more frequently contributing to an observed correlation of degree of homophily and strength of the tie between individuals [32]. Moreover, the initial similarity produces a contagion effect where actors adapt their behaviour and beliefs to match those around them [32,33]. Homophily has been found in healthcare settings based on such variables as profession, [34,35] gender [35], and key tasks [36].

A parallel concept from the management literature on interorganisational collaboration is that of “proximity”. Like homophily, the degree of proximity also affects the formation of clusters and the efficiency of network functions. Proximity may be geographic, reflecting the physical degree of closeness, or organisational, reflecting similarity in thinking styles, training or educational background, competencies, shared experiences or common informal knowledge [10,17].

Trust and reputation are two other factors strongly influencing the emergence of collaborative network ties. Work by Gulati and colleagues have shown that collaborative interorganisational alliances are strongly influenced by trust generated in previous partnerships, and by reputation mediated by a third partner [37].

We developed five hypotheses around factors influencing the pattern of ties present before the TRN began, and the nature of current and future intended collaborations as part of network activities. Each hypothesis is predicated on testing how three literature-derived factors associated with clustering affect collaboration, i.e. geographic proximity, professional proximity, and past experience.

### Geographic proximity

Geographic proximity (e.g. working in the same hospital, or campus) [35] allows enhanced opportunities to interact and can result in clusters for certain types of interaction. It facilitates face-to-face interactions, whether planned or not, which in turn support knowledge transfer and sharing of ideas [17,38,39]. The TRN is spread across multiple campuses and opportunities for interactions between organisations had been limited before the formation of the network. Thus the first hypothesis stated:

**Hypothesis 1:** Collaborations are more common among members working at the same site.

### Professional proximity

Organisational proximity describes a sharing of routines, cultures, values and norms allowing the ready interpretation and understanding of transferred knowledge from one member to another [17]. Both the member’s professional education and the type of institution in which the member works have generic features in common. For instance, any surgeon would typically have a closer understanding of routines and practices of any other surgeon than of a protein chemist. Research paradigms, norms and *modus operandi* differ significantly between researchers and clinicians making them less likely to collaborate [40]. Thus we derived hypothesis two:

**Hypothesis 2:** Collaborations are more common between two or more researchers, or two or more clinicians, than between clinicians and researchers.

### Past collaborative ties

Other factors expected to influence patterns of collaboration are previous working relationships and the strength of those ties. People who have collaborated previously are more likely to collaborate again as they will have had the opportunity to develop trust, shared understandings and values, and the strength of those ties are more likely to be reported as strong or very strong [16]. Therefore we would expect to see the majority of current and future collaborations reported between people who have previously worked or collaborated together and have strong or very strong relationships, leading to our next hypothesis:

**Hypothesis 3**: People who have collaborated together in the past and report strong ties between them are more likely to be collaborating now and intending to collaborate in the future.

### Past translational research experience

The likelihood of members being actively involved in the network may also be increased by their previous experience of translational research making them more likely to report collaborations now and in the future. The desire to undertake more of this research may be a motivating factor [41,42]. As a result, we proposed hypothesis four:

**Hypothesis 4:** People who have past experience of translational research are more likely to be involved in current and future collaborations.

### Collaborative intentions

Networks aim to increase opportunities to interact with people to whom members previously may have had no access. People previously known only through reputation (heard them speak at a conference, or read their published research) or an indirect contact (e.g. a colleague of a colleague) may now be accessible for collaboration through the structure of the network. If this process is happening, we would expect future intended collaborations to feature more of these weak ties than were reported prior to the network or in the first round of project negotiations. Reported future intended collaborations that were made up solely of strong past ties would suggest business as usual; *i.e*. that the network had not changed or expanded existing collaborative partnerships. Hence our final hypothesis:

**Hypothesis 5:** Future intended collaborations have the most ties reported as weak or very weak, or based on a member’s reputation compared to past or current collaborations.

## Methods

People listed as full members of the TRN as of January 2012 were asked to complete an on-line survey in March 2012, each member receiving a link to the secure survey site via personal email. Ethics approvals were obtained from the University of New South Wales (HREC: 09085) and appropriate local health network and site-specific committees. Respondents were assured of anonymity in the reporting of results (names being replaced by anonymous codes) and were required to give formal consent.

The survey was informed by interviews with 14 network stakeholders and feedback from a pilot of the survey by ten participants from equivalent clinical or research backgrounds. The survey was a whole network survey [43,44], that is we sought answers from all members to reflect the whole network, rather than a sample of members. To maximise the response rate, three follow-up reminders were emailed to non-respondents over the next two months.

The survey established respondents’ place of work, main tasks, years of experience, and if they had had previous involvement in translational research. Workplaces were grouped into three sites - Central, Satellite and Peripheral - based on both geographic proximity and professional proximity; i.e. having administrative links (largely NSW Local Health districts). The Central Site grouped six of the workplaces which were all within a one-kilometre radius of each other. The Satellite Site grouped five workplaces which were close to one another but 16 kilometres from the Central Site. The Peripheral Site grouped the remaining seven scattered workplaces that had no administrative or organisational links with the Central Site. Each respondent was categorised as a “clinician”, “researcher”, or “clinician-researcher” based on their reported key tasks, place of work and preferred title.

The second section of the survey asked social network questions (Table 1). Each question provided a roster of members’ names, job titles and primary place of work as an aid to memory. Past, Current and Future intended network graphs (sociograms) were constructed from these questions (i.e. each question produced a separate sociogram with the same respondents as the nodes but different configurations of ties between them) and the type of tie (e.g. work colleague, known by reputation) and current strength of the relationship was also given. Questions sought to differentiate between a working relationship (e.g. shared care of patients or worked in the same lab) which may involve little individual choice of partners, and collaborative relationships (e.g. co-investigators on a funded project, participating in a clinical trial or audits) which involve negotiation and choice. Strength of tie sought to quantify the tie in some way as working with someone once in 1982 but not again cannot be compared with someone who has worked continuously with someone else over the last two years. The category “strong, enduring relationship” was included to describe situations such as in medical training where a strong mentoring relationship early in one’s career pervades all future work regardless of frequency of contact. Feedback from the pilot survey with clinicians and researchers from equivalent contexts found this an intuitive and useful category.

**Table 1:** Social network survey questions

**A full list of members’ names, job title and primary workplace was provided for each question *e.g Jane Doe, *** **Nurse, Hospital #1; *John Smith, *** **Research Fellow, University A.**	
**Past collaboration network**	
*Q1. Work your way down the list of members and select the description that best fits that person: (type of tie)*	*Q2. Select the current strength of relationship with the people you say you know (Strength of tie)*
1. I do not know this person at all	a) Very weak: I hardly know this person or I used to know them but we no longer stay in contact
2. I know this person through collaboration on a research project before the TRN began (e.g. clinical trials, NH&MRC funded projects, quality improvement projects, audits)	
	b) Weak: we have infrequent and/or superficial contact
3. I have worked or liaised with this person in another way, including current work colleagues (E.g. shared care of patients, worked in same lab, shared resources)	
	c) Strong: we have frequent and/or purposeful contact
4. I know this person by reputation only (e.g. read their paper, heard them speak, colleague of a colleague)	
	d) Long-term: this is a long-term, enduring relationship regardless of the frequency of contact
**Current collaboration network**	
*Q3. Work your way down the list of members and select any that fit the following description:*	
I have consulted or collaborated with this person regarding any TRN research project or have collaborated with them regarding dissemination of TRN objectives or findings.	
**Future intended collaboration network**	
*Q4. Work your way down the list of members and select any that fit the following description*	
I would consider collaborating with this person on a TRN project in the future	

Social network answers were analysed using UCInet v6 [45] to determine each sociogram’s characteristics and to determine the influence of the two grouping categories on patterns of clustering. We measured density, number of network components, External-Internal (E-I) indices, clustering coefficients, and network diagrams were generated in NetDraw [46]. E-I index measures the number of ties within a defined group compared to the number of ties to outside of the group and tests for a pattern of links compared to links made randomly (α = 0.05). This shows whether the grouping category has an influence on ties or not. Categories considered were site (geographic proximity), and professional group (professional proximity). Clustering coefficients average the densities of each member’s immediate contacts and when compared with overall network densities can give another network level indication of local clustering. Comparisons were also made across Past, Current and Future Collaboration sociograms considering members’ past involvement in translational research, and strength and type of tie.

## Results

The response rate for the survey was 76.5% (52/68). A comparison of respondents and non-respondents showed that they were similar in gender distribution (*χ*^2^ (1, n = 68) 1.85, p = 0.17) and representation from Central, Satellite and Peripheral Sites (*χ*^2^ (2, n = 68) 5.27, p = 0.072). The lowest response rate came from the peripheral sites.

Sixty-three per cent of respondents worked at a Central site, 27.8% of members at a Satellite site and 9.2% were at a Peripheral site. Thirty eight per cent of respondents were classified as clinicians, 53.7% researchers and 7.4% clinician-researchers with joint clinical and academic positions. Based on survey responses, most members had been involved in translational research in the past (80.8%).

**Figure 1:**
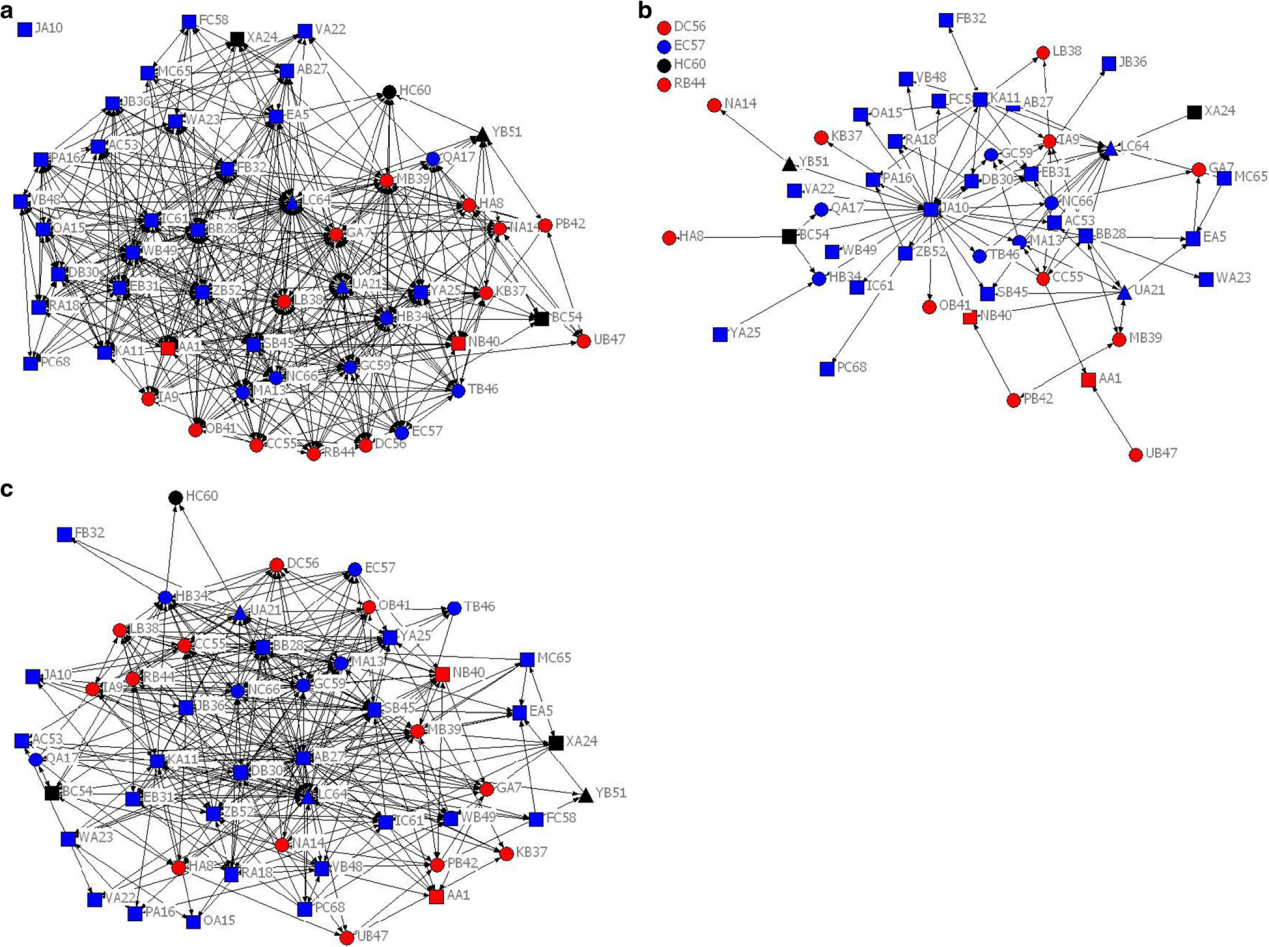
**Past, current and future collaboration networks. (a)** Past collaboration network. Each node represents a survey respondent and each line a tie defined by the questions: “I know this person through collaboration on a research project before the translational research network began (e.g. through clinical trials, NHMRC funded projects, quality improvement projects, audits)” or “I have worked with this person in another way, including current work colleagues (e.g. shared care of patients, worked in the same lab, shared resources).” TRN director is LC64; JA10 is the TRN manager. Square nodes are clinicians, circles are researchers and triangles are clinician-researchers. Blue are members from a Central Site, red are from a Satellite site and black from a peripheral site. **(b)** Current Collaboration network. Links defined by the question: “I have consulted or collaborated with this person regarding a translational research network project or regarding dissemination of its objectives or findings.” Four respondents are not currently collaborating. JA10 is the TRN manager. **(c)** Future Collaboration network. Links defined by the question: “I would consider collaborating with this person on a translational research network project in the future.”

Sociograms are shown in Figures 1(a) – (c) and their parameters are summarised in Table 2. There was a difference in the way respondents interpreted the social network questions shown by only 39.7% reciprocity for the question “With whom have you collaborated” and 60.1% for the question “With whom have you worked”. Reciprocity measures whether ties are mutually acknowledged: a reciprocated tie is one in which A nominates B as a tie and B nominates A. For collaboration and working ties we expected this to be much higher. Aggregating collaboration ties with the work ties lifted reciprocity to 80.2%, so the Past Collaboration sociogram was constructed by combining these two ties. The Past Collaboration sociogram contained 990 ties with every respondent nominating between six and 49 other members. Fifty-one per cent of these ties were described as weak or very weak and 49% were described as strong or very strong.

**Table 2:** Whole network parameters for the three sociograms: past, current and future collaboration

**Parameter**	**Past collaboration**	**Current collaboration**	**Future collaboration**
*Total number of ties reported*	826	106	395
*Number of respondents reporting ties*	52	26	46
*Number of isolates*	1	4	0
*Network density*	0.312	0.040	0.149
*Network clustering coefficient (weighted)*	0.492	0.106	0.269
*Network components*	2^a^	5^b^	1
*E-I Indices:*			
*Geographic proximity (work site)*	-0.208^c^	-0.426^c^	-0.209^c^
*Professional proximity (professional group)*	-0.115^c^	0.000	0.012

^a^One major component and 1 isolate.

^b^One major component and 4 isolates.

^c^Indicates statistically significant association.

A pattern of clustering was demonstrated across the Past Collaboration, Current Collaboration and Future Collaboration sociograms. Clustering coefficients for each were higher than their respective network-wide densities indicating areas of higher densities within groups. There were no exclusive groups unlinked to others: component analysis showed a single component in all three sociograms (not counting isolates as groups or components here).

### Bases of clustering

Geographic proximity (work site) was found to influence collaborative ties with significant, negative E-I indices (shown in Table 2) indicating that there were more past, current and future collaborations among members working at the same site, than to other sites, thus supporting Hypothesis 1.

Professional proximity (professional group) of the member also influenced collaborative ties in the Past Collaboration sociogram. Professional group did not significantly influence ties in the Current and Future Collaboration sociograms, thus giving only partial support for Hypothesis 2.

**Figure 2:**
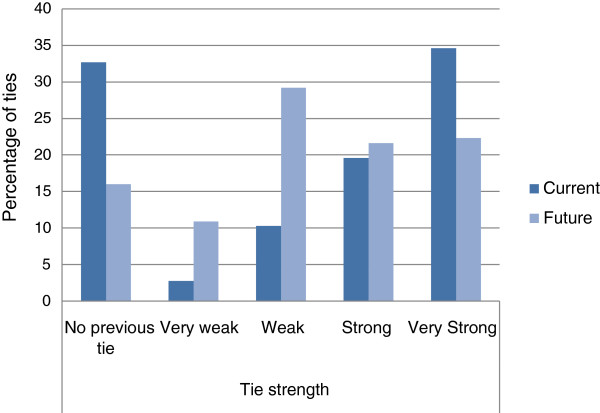
**Percentage of ties by strength for current and future collaboration sociograms.** All except one of the Current collaboration ties between members that had no tie previously are from and to the TRN manager.

The majority of respondents in the Current and Future Collaboration sociograms had worked or collaborated in the past, providing support for Hypothesis 3. In the Current Collaboration sociogram, 67.3% of all ties were between people who had worked or collaborated before the TRN had formed, and considering the strength of ties, 54.2% of all ties were reported as strong or very strong (see Figure 2). Ties to and from the TRN manager alone accounted for all but one of the other 32.7% of the total ties. The TRN manager knew three members by reputation before the TRN formed. The remainder were unknown to her. In the Future Collaboration sociogram 78.2% of ties were between members who had collaborated or worked together in the past and 43.9% were strong or very strong ties. There were 16.0% of ties that were not based on previous working relationships; the TRN manager did not nominate any of these future intended ties.

Forty-two respondents had experience of translational research while ten did not. The majority (88.5%) of Current Collaborations were reported by members who had past experience of translational research. Only three of the ten members who had not been involved in translational research reported current collaborations. However nine of the ten with no experience reported future intended collaborations. Thus Hypothesis 4 is supported in the Current Collaboration sociogram but not supported in the Future Collaboration sociogram.

Ties described as very weak (“I hardly know this person or we no longer stay in contact”) changed from 2.7% in the Current to 10.9% in the Future Collaboration sociograms, supporting Hypothesis 5. There were also more ties with people that were known only through reputation in the Future Collaboration sociogram (5.8% of ties) than in the Current Collaboration sociogram (3.6%) supporting Hypothesis 5. Apart from the ties involving the TRN manager, ties to people they did not know at all before the TRN started also rose from 0.9% (one tie from a single member) in the Current sociogram to 16% (63 ties from 10 members) in the Future sociogram.

## Discussion

A summary of the hypotheses and the findings is presented in Table 3. All three of the collaboration sociograms showed patterns of collaboration based on one or more factors. Past Collaborations were significantly influenced by geographic and professional proximity. The Current and Future Collaboration sociograms were significantly influenced by geographic proximity and past collaborative ties.

**Table 3:** Summary of hypotheses and findings

**Hypotheses**	**Findings**
**Hypothesis 1:** Collaborations are more common among members working at the same site.	**Supported** across Past, Current and Future Collaborative tie networks.
**Hypothesis 2:** Collaborations are more common between two or more researchers, or two or more clinicians, than between clinicians and researchers.	**Partially supported.** This was true of Past Collaborations but not for Current and Future intended Collaborations.
**Hypothesis 3:** People who have collaborated together in the past and report strong ties between them are more likely to be collaborating now and intending to collaborate in the future.	**Supported.** Current Collaboration ties: 54% were described as strong or very strong and 67.3% had worked together or collaborated before. The TRN manager accounted for the other 32.7% of ties.
	Future ties: 43.9% were described as strong or very strong and 70.6% had worked together or collaborated before.
**Hypothesis 4:** People who have past experience of translational research are more likely to be involved in current and future collaborations.	**Partially supported.** Only 10 of the 52 respondents reported no experience. The Current Collaboration network had 88.5% of members with past experience and only three of the 10 without experience. In the Future intended network there were nine of the ten with no experience.
**Hypothesis 5:** Future intended collaborations have the most ties reported as weak or very weak, or based on a member’s reputation compared to the past or current collaborative networks.	**Supported.** There were 2.8% ties described as weak in the Current Collaboration network while there were 29.3% described as weak in the Future intended network. Ties that were known by reputation rose from 3.8% in the Current to 9.4% in the Future intended network. Members that were described as not known at all went from one member reporting one tie in the Current to 10 members reporting 64 ties in the Future intended network.

Working at the same hospital or university does not necessarily mean the individuals are known to each other or that they have anything other than familiarity with the organisation in common. The influence of geographic proximity however is seen across all three collaboration sociograms. This likely reflects the practical incentive of choosing a partner who is close at hand: face-to-face meetings are easier to arrange and the will to collaborate may be fostered through serendipitous meetings in the corridor, car park or cafeteria [17]. The difficulty of transferring complex knowledge or maintaining momentum on a project is harder when geographic distance limits frequency of access. This has implications for a geographically dispersed network such as the TRN, identifying opportunities for more collaboration if strategies to bridge sites are addressed. Difficulties in running geographically dispersed collaborations include the need for adequate technology for virtual communication (e.g. teleconferences or video links), adaptation to this form of communication where social cues such body language and tone of voice can be lost or hidden, and an altered process of building trust between partners [16]. Social interaction apart from actual project negotiations have been shown to be useful in building long distance trust [47] so the inclusion of social gatherings in the TRN calendar of events would be recommended. The strengths of same site collaborations can be fostered through ready local access to resources at each of the sites.

The decrease in the within professional group ties from Past to Current and Future sociograms suggest that more members are currently taking advantage of, or are intending in the future to take advantage of, the opportunities the TRN structure provides for interdisciplinary collaboration. This is a significant finding as the gap between clinicians and researchers is seen as a major barrier in the translational research literature [40,48].

The majority of collaborations in the Current and Future sociograms were with members who had a pre-existing working relationship. Considering the ties in the Current Collaboration sociogram that were between members with a pre-existing working relationship only 52.4% were described as strong or very strong meaning some of the pre-existing relationships that were being renewed were previously weak or very weak. The suspicion is that the structure of the network has allowed the renewal of old, weak ties and has bridged some gaps.

The number of Future intended collaborations reported was very encouraging and showed an enthusiasm for TRN activities. The number of intended ties based on reputation, or ties described as very weak or non-existent before the formation of the network were also reasons to be encouraged that the network was increasing connectivity between members that had no previous working relationship. Reminding members of their future intended collaborations after some time has elapsed and enthusiasm has waned may reinforce and realise those intentions.

People may choose to collaborate if they recognize that the other person holds expertise or access to resources that could assist them [49,50] but learning what expertise or resources are available among members, often held collectively in clusters, can be difficult. TRN email and website features are one source of information but another is personal introduction via a go-between or broker [51]. The key roles of the TRN director and TRN manager as central actors and brokers, co-ordinating and linking together members is investigated further in separate studies [13-15]. The star–shaped pattern of the sociograms [52], most evident in the Current Collaboration sociogram (Figure 1(b)) shows the strong influence that one or two actors can exert on a network and how vulnerable the TRN is to their loss. The TRN director had by far the most ties to other members in the Past Collaboration sociogram while the TRN manager dominates the Current Collaborations. She had systematically contacted all members and visited or interacted with over half of the membership at the time of the survey. These two members, having knowledge of the expertise of most members, are in an ideal network position to broker new collaborations. The increase in future intended collaborations between members known by reputation or having only very weak ties previously shows that members are reaching beyond their familiar associates. Whether this is through the brokerage activity of the TRN director and manager is not clear. Future surveys will show whether intended ties become actual collaborations and whether projects bear fruit.

While this study has focused on just one translational research network, albeit a complex one, our results have relevance to many similar networks and alliances that have been founded to address the research–practice gap: for example the Collaboratives for Leadership in Applied Health Research and Care (CLAHRCs) in the UK [53,54] and the interorganisational alliances awarded funded by the Clinical and Translational Science Awards in the USA [28,55-57]. Many face similar issues of bridging cultural and epistemic differences between researchers and clinicians and facilitating geographic distance.

### Limitations and strengths

Social network surveys can provide a wealth of data but can also raise methodological issues. The whole network approach, where the survey is a census not a sample, promises a comprehensive network picture but can be difficult to achieve, especially when, as here, our contact with non-responders was limited to email. Our response rate was over 76% meaning that 24% of the TRN members had not stated their ties. There does not seem to be a consensus in the published literature on valid response rates of whole network surveys. A discussion on Borgatti’s social network forum suggests the key considerations in judging validity are the robustness of the measures, the structure of the network, and the nature of the ties, i.e. can the missing data be imputed from the available data [58]. By these standards, the dense network structure of the TRN, with high reciprocity and an acceptable match of demographic features between respondents and non-respondents, we were confident that 76.5% was valid. A factor that is unknown is whether the respondents of the survey are also the members most likely to collaborate and therefore have skewed results.

To maximise the accuracy of the respondents’ self-reported ties we chose a roster format where each member was listed by name, job title and place of work rather than a name generator where respondents are asked to just list all their contacts. While the roster of names solves the problem of imperfect recall of who you might know, it is quite a lengthy task for respondents to work through all the names and allocate to each, one of four categories of ties. In the present study only two respondents did not complete all the social network questions, one citing internet display issues as the reason rather than “respondent fatigue”.

While memory and respondent fatigue may lead to under reporting of existing ties, self report may carry a risk of over-reporting. Collaborative ties are seen as socially desirable, especially so in this network context, so respondents could have exaggerated their links to others. Reciprocity testing however, showed that 80.2% of work and collaborative ties in the past came from both partners confirming the majority of links and suggesting that this is not a problem.

This study fits into a larger program of research on networks [13] and repeats of the survey at a later date will enable a longitudinal view of changes and growth in collaborative activity. The role of brokers and central actors in facilitating collaborative linkages will also be examined.

## Conclusions

Diverse groups of people are brought together in healthcare networks in order to maximize cross-fertilization of ideas, transfer of knowledge, co-ordination of activities and access to resources. There are both strengths and opportunities to be found in clusters. Cohesive groups can function as highly productive work teams, holding and developing a store of knowledge and expertise. However, to prevent such clusters becoming islands, the gaps between them need to be recognised and ways found to bridge them. Research on this TRN has shown that patterns of collaboration are based on clustering by geographic proximity and previous collaborations. Future intended collaborations include a significant number of weak ties and ties based on other members’ reputations implying that the TRN has provided opportunities for partnership that may not have been available before. The highly centralised shape of the Current Collaboration sociogram suggests co-ordination and mediation by key network players. Geographic proximity remains a significant influence on choice of partner perhaps reflecting members’ preference for working locally.

## Competing interests

The authors declare that they have no competing interests.

## Authors' contributions

Protocol developed by JCL, FCC, PC and JB. JCL wrote the paper and FC, PC and JB critically reviewed all drafts and final copy. All authors read and approved the final manuscript.

## Pre-publication history

The pre-publication history for this paper can be accessed here:

http://www.biomedcentral.com/1472-6963/14/225/prepub
